# Organic Extract of* Justicia pectoralis* Jacq. Leaf Inhibits Interferon-γ Secretion and Has Bacteriostatic Activity against* Acinetobacter baumannii* and* Klebsiella pneumoniae*

**DOI:** 10.1155/2018/5762368

**Published:** 2018-08-23

**Authors:** Tiago Rafael de Sousa Nunes, Marina Ferraz Cordeiro, Fernanda Gomes Beserra, Matheus Landim de Souza, Wliana Alves Viturino da Silva, Magda Rhayanny Assunção Ferreira, Luiz Alberto Lira Soares, Sérgio Dias Costa-Junior, Isabella Macário Ferro Cavalcanti, Maira Galdino da Rocha Pitta, Ivan da Rocha Pitta, Moacyr Jesus Barreto de Melo Rêgo

**Affiliations:** ^1^Laboratory of Immunomodulation and New Therapeutical Approaches, Research Centre for Therapeutic Innovation-SuelyGaldino (NUPIT-SG), Federal University of Pernambuco, Recife, PE, Brazil; ^2^Department of Medicine, Federal University of the São Francisco Valley (UNIVASF), Paulo Afonso, Brazil; ^3^Laboratory of Pharmacognosy, Department of Pharmaceutical Sciences, Federal University of Pernambuco, Recife, PE, Brazil; ^4^Laboratory of Immunopathology KeizoAsami (LIKA), Federal University of Pernambuco, Recife, PE, Brazil; ^5^Microbiology and Immunology Laboratory of Academic Center of Vitoria, Federal University of Pernambuco, Recife, PE, Brazil

## Abstract

*Justicia pectoralis* Jacq. (Acanthaceae) leaves currently found in the Brazilian north-east are widely used to treat diabetes, menstrual pains, asthma, and other disorders. This work aimed to identify the phytochemical characterization and biological activities of* J. pectoralis* leaf extracts. The plant material was ground and the crude extracts were obtained with water or acetone: water (7:3 v/v), yielding aqueous (JPA), and organic (JPO) extracts. Phytochemical characterization was performed by thin-layer chromatography (TLC) and high-performance liquid chromatography (HPLC). Cytotoxicity was assessed by 3-(4,5-dimethylthiazol-2-yl)-2,5-diphenyltetrazolium bromide) (MTT) assay and trypan blue (TB) exclusion assay in peripheral blood mononuclear cells (PBMCs), BALB/c splenocytes, and neoplastic cells (TOLEDO, K562, DU-145, and PANC-1) at 1, 10, and 100 *μ*g/mL. Antibacterial activity was evaluated using the microdilution test to obtain the minimum inhibitory concentration (MIC) and minimum bactericidal concentration (MBC). Cytokines, IFN-*γ*, and IL-17A from culture supernatants of BALB/c mice splenocytes were measured by sandwich ELISA. In the TLC analysis, both JPA and JPO extracts presented coumarin and flavonoids. In addition, HPLC was able to identify coumarin, apigenin, and ellagic acid in both extracts. JPO IC_50_ was 57.59 ± 1.03 *μ*g/mL (MTT) and 69.44 ± 8.08 *μ*g/mL (TB) in TOLEDO. MIC value of JPO against* Acinetobacter baumannii* and* Klebsiella pneumoniae* was 500 *μ*g/mL. JPO (100 *μ*g/mL) significantly inhibited IFN-*γ* levels (p=0.03).* J. pectoralis* is a potential candidate to be further investigated as an IFN-*γ* inhibitory agent and against* Acinetobacter baumannii* and* Klebsiella pneumoniae*.

## 1. Introduction

*Justicia pectoralis *Jacq. belongs to the family Acanthaceae [1] and in Brazilian north-east it is popularly known as chambá, chachambá, anador, clover-tree, or clover-cumaru. It is quite widespread, and its occurrence has been registered in several South American countries [2]. There are also reports of its traditional uses. One of the first publications was a report by MacRae and Towers [3] who pointed out its administration against lung infections, stating it to be an ingredient of “hallucinogenic” snuff. In the form of syrups, infusion, or mash, it was found that the leaves of this plant were used as an expectorant and to treat asthma, cough, bronchitis [2, 4], colds, menstrual pains [5], and diabetes and as an antibacterial and sedative agent [6].

Other studies indicate that aerial parts are also used against nervousness and sleeplessness, as a hypotensive [7, 8], and against epilepsy [9]. However, few authors have described its antineoplastic activity, with most studies about this plant concentrating on its use for treatment of respiratory tract disorders and consequently its anti-inflammatory potential [10]. Some of these anti-inflammatory studies include clinical trials with* J. pectoralis* syrups for treatment of asthmatic patients [11–13]. However, only one study has reported the cytokines modulation, showing exclusively the modulations of IL-1*β* and TNF-*α* cytokines [2].

Regarding the types of extract, most researchers have used aqueous or hydroalcoholic extracts. However, the most efficient extraction of phenolic compounds is acquired using an organic solvent such as acetone [14]. A range of biological activities performed by medicinal plants are due to the presence of phenolic compounds and other phytochemicals [15, 16] and, in this sense, acetone was the organic solvent used in this work to extract a high amount of these compounds. Thus, considering the traditional knowledge and the use of organic solvents that extract a high number of compounds, the present study aimed to explore the biological activities of the aqueous and organic extract of* J. pectoralis *leaves.

## 2. Materials and Methods

### 2.1. Plant Material and Extracts Preparation

Specimens were collected at the Training Center of the Agricultural Research Institute (CETREINO/IPA), located in Carpina, Pernambuco, Brazil (07° 51′ 03′′ S 35° 15′ 17′′ W), under controlled growth conditions. After collection, a voucher specimen (# 91413) was identified and deposited in the IPA Herbarium. Afterwards, the leaves were removed and dried in an oven for 48 hours. The dried material was then milled using a knife mill (TE-680, Tecnal). The extracts were obtained by 10% (w/v) turbo-extraction (Metvisa) using water or an acetone:water mixture (7:3, v/v). The extracts were filtered by vacuum and the organic extract was concentrated under reduced pressure (RV10 Basic, IKA) to remove the acetone. The filtrates were frozen (3 days, T = -80°C) and the remaining solid material was lyophilized for 48 hours (L101, Liotop) to yield the aqueous extract (JPA) and organic extract (acetone:water - JPO) from* J. pectoralis*.

### 2.2. Phytochemical Characterization by TLC

The crude extracts were dissolved in methanol and agitated in a vortex (LabDancer, IKA) to complete solubilization, obtaining a final concentration of 1 mg/mL. For thin-layer chromatography (TLC) screening, all standards were used at 1 mg/mL. The samples and standards were applied, 20 *μ*l and 5 *μ*l, respectively, on silica gel 60 - F_254_ (Macherey-Nagel) chromatographic plates, using a semiautomatic applicator (Camag) and software (WinCats). The chromatographic plates were developed in a twin-trough glass chamber (20 cm x 10 cm, Camag) after saturation with the mobile phase (Supplementary Table S1) for about 30 minutes at room temperature (25 ± 2°C).

The bands were applied with a width of 10 mm and a distance between them and the edges of plates of 5 mm. The width and length of the chromatographic plates were 5 cm and 10 cm, respectively. The samples were applied at 5 mm from the origin and with 5 mm from the end of the plate. After elution, the plates were dried at room temperature and observed under ultraviolet light of 254 and 365 nm and visible light. Then, the plates were derivatized with reagents specific for each metabolite (Supplementary Table S1). The bands visualized on the samples were compared to bands of the corresponding standards.

### 2.3. High-Performance Liquid Chromatography (HPLC) Analysis

JPA and JPO (5 mg) were weighed and transferred to a volumetric flask (5 mL) with ultrapure water (Purelab Classic UV, Elga). The samples were brought to the ultrasonic bath (Ultracleaner, Unique) for 15 minutes for complete dissolution. The volume was then completed with ultrapure water. After dilutions, the samples were filtered into vials through a PVDF membrane (25 mm, 0.45 *μ*m).

The analysis was conducted in an HPLC (Ultimate 3000, Thermo Fisher Scientific) equipped with photodiode array detector (PDA–3000 [RS]; Thermo Fisher Scientific), a binary pump (HPG3x00RS, Thermo Fisher Scientific), a degasser, and an autosampler. The analysis was performed in a C_18_ column (250 mm x 4.6 mm, 5 *μ*m; NST) equipped with a precolumn C_18_ (4 mm, 3.9 *μ*m; Phenomenex), maintained at 24°C. The mobile phase consisted of purified water as solvent A and methanol as solvent B, both acidified with trifluoroacetic acid (0.05%), with the flow adjusted at 0.8 mL/minute. Both were degassed in an ultrasonic bath and filtered through a membrane (45 mm, 0.45 *μ*m). The injection volume used was 20 *μ*l. The wavelength of the analyses was set at 280 nm. The separation was conducted using the following gradient: 0-10 min, 15-30% B; 10-20 min, 30-50% B; 20-25 min, 50-75% B; 25-28 min, 75-15% B; 28-32 min, 15% B. The standards coumarin (98% of purity), apigenin (analytical standard), and ellagic acid (96% of purity) were purchased from Sigma-Aldrich (São Paulo, Brazil). The experiments were performed in triplicate.

### 2.4. Cytotoxicity Evaluation

Cytotoxicity was initially evaluated in nontransformed cells (peripheral blood mononuclear cells [PBMCs] from healthy donors and Balb/c mice splenocytes) and then in neoplastic cell lines. Both evaluations were performed with MTT (3-(4,5-Dimethylthiazol-2-yl)-2,5-Diphenyltetrazolium Bromide) assay according to Carvalho et al. [17] and with the trypan blue exclusion method. The assays were only initiated after approval from the Human Ethics Committee CAAE: 46976315.9.0000.5208 and the Ethics Committee for the Use of Experimental Animals of the UFPE (process number 23076.041556/2015-62). PBMCs were used from four clinically healthy individuals who did not meet the exclusion criteria. These criteria included individuals that had ingested alcohol in the last 72 hours or were taking any medication. After signing the Term of Free and Informed Consent, blood was collected in heparin tubes. PBMCs were isolated by centrifugation with FicollPaque Plus (GE Healthcare Bio-Sciences) at 350 g for 45 minutes and collected in the intermediated white phase where PBMCs are commonly found. Afterwards, they were cultured in RPMI 1640 medium (Gibco) supplemented with L-Glutamine, 10% fetal bovine serum (Lonza), 10 mM HEPES (Gibco), and 200 (4-(2-hydroxyethyl)-1-piperazineethanesulfonic acid) U/mL Penicillin/Streptomycin (Gibco). PBMCs were used only when they showed viability equal to or greater than 98% after counting in a Neubauer chamber with trypan blue reagent [18].

PBMCs were grown in a 5% CO_2_ incubator at 37°C in the amount of 5.5x10^5^ cells/well in quadruplicate for 48 h with JPA and JPO at 0.1 to 100 *μ*g/mL. JPA was diluted using only culture media and DMSO was used to dilute JPO. After 48 h, MTT reagent (20 *μ*g/mL) was added and subsequently analysed [17]. The analysis was performed using a cell viability formula (1), as described below:

(1) VMTT%=MC−MBextract×100MC−MBRPMI  or  DMSO,

where MC is the mean of triplicate of cell well and MB represents the mean of triplicate of blank well.

To evaluate the cytotoxicity with trypan blue exclusion assay, after incubation for 48 h with JPA and JPO at 0.1, 1, 10, and 100 *μ*g/mL, a 20 *μ*l aliquot of the cell suspension was diluted in 20 *μ*l of trypan blue. The cells were observed for their morphological changes and counted in a Neubauer chamber (blue cells were considered to be dead). After counting, the following mathematical formula (2) was applied, which gives the result of the cytotoxicity of the extracts against PBMCs through the cell viability result of each condition:

(2) $$\begin{array}{c} V_{TB}\left(\;\%\;\right)=\left[\;\left(\;\frac{live\;\;cells}{\left[live+dead\;\;cells\right]}\;\right)\times 100\;\right] \end{array}$$

As a negative control, the condition with only cells was used for the diluted extract in a serum-free medium (JPA). The condition of cells with 0.1% DMSO was used for the extract diluted in DMSO (JPO). The same methodology was employed to evaluate the cytotoxicity in neoplastic cell lines: TOLEDO (B cells Lymphoma), K562 (Chronic Myeloid Leukaemia), DU-145 (Prostate Cancer), and PANC-1 (Pancreatic Cancer) at 1, 10, and 100 *μ*g/mL. However, cells were plated according to each doubling time in accordance with NCI60 guidelines cells/well and incubated for 72 h. At 0 h of treatment, K562 cells were at a density of about 5x10^3^ cells/well and TOLEDO, DU-145, and PANC-1 at 1x10^4^ cells/well. Amsacrine, doxorubicin, or gemcitabine was used as a positive control.

To evaluate cytotoxic profile of JPA and JPO in BABL/c splenocytes, animals were obtained from LIKA/UFPE. The mice were sacrificed in a CO_2_ chamber, according to the Guidelines of the Ethics Committee for the Use of Experimental Animals of the UFPE. Each spleen was aseptically extracted and placed in a petri dish containing RPMI-1640 (Gibco) to obtain splenocytes. The obtained cell suspension was filtered on 40 *μ*m nylon (BD Falcon) and transferred to Falcon tubes. Spleen concentrates were centrifuged twice for 10 minutes. Subsequently, cells were lysed with 1X red blood cells (RBC) lysis buffer (eBiosciences) and resuspended in RPMI-1640 medium (Sigma) supplemented with 10% fetal bovine serum, 10 mM HEPES (4-(2-hydroxyethyl)-1-piperazinoethanesulfonic acid) (Gibco) and 200 U/mL penicillin/streptomycin (Gibco). The evaluation of JPA and JPO cytotoxicity was performed by incubation (10 and 100 *μ*g/mL) for 48 h at 5% CO_2_ and 37°C. After 48 h, MTT and trypan blue were added as described above.

### 2.5. Antibacterial Activity

*Acinetobacter baumannii*, extended-spectrum beta-lactamase-producing Klebsiella pneumoniae (ESBL), and* Klebsiella pneumoniae *carbapenemase (KPC) clinical isolates were obtained from the Clinical Hospital of the Federal University of Pernambuco and kept at the Laboratory of Immunopathology Keizo Asami (LIKA/UFPE). These strains were phenotypically identified according to the guidelines of the Clinical and Laboratory Standards Institute (CLSI) [19]. Methicillin-resistant* Staphylococcus aureus* (ATCC 33591), methicillin-sensitive* Staphylococcus aureus* (MSSA) ATCC 29213,* Escherichia coli *ATCC 25922,* Klebsiella pneumoniae* ATCC 29665, and* Pseudomonas aeruginosa* ATCC 27853 were used as reference strains.

The antibacterial activity of JPA and JPO was determined by microdilution test according to CLSI [19]. Microdilution plates (96-well) were filled with Müller-Hinton broth (MHB) and the extracts previously diluted in 0.5% DMSO were distributed through serial dilution to obtain concentrations ranging from 1 to 500 *μ*g/mL. Vancomycin (VAN) and Ciprofloxacin (CIP), at concentrations ranging from 0.0075 to 3.84 *μ*g/mL, were used as reference drugs for Gram-positive and Gram-negative bacteria, respectively. Subsequently, bacterial suspensions were adjusted to 0.5 of the McFarland scale and diluted to a final concentration of 10^5^ CFU/mL in each well. Microplates were incubated at 35 ± 2°C for 24 h and minimum inhibitory concentration (MIC) was determined by spectrophotometric analysis at 620 nm. MIC was defined as the lowest extract concentration that inhibited bacteria growth >90%. Minimum bactericidal concentration (MBC) was determined in subculture samples from wells with concentrations above MIC in plates containing Müller-Hinton agar (MHA). These plates were incubated at 35 ± 2°C for 24 hours and MBC was considered the lowest concentration of extracts associated with absence of bacterial growth. All experiments were performed in triplicate.

### 2.6. Immunomodulatory Activity

To evaluate the proinflammatory cytokines modulation, eight male BALB/c mice were used with 45 days. After cytotoxicity evaluation, splenocytes were cultured in 24-well plates (2x10^6^/mL in each well) with RPMI-1640 medium (Gibco) supplemented with 10% fetal bovine serum (Gibco), 10 mM HEPES (Gibco), and penicillin and 200 U streptomycin/mL (Gibco). As stimuli, Concanavalin A (ConA) was used at 100 ng/mL. As the reference drug, methylprednisolone (MP) was tested at 100 *μ*M. JPA and JPO extracts were added at concentrations of 10 *μ*g/mL and 100 *μ*g/mL and incubated at 37°C and 5% CO_2_ for 48 h. After the incubation time, 1 ml of culture supernatant was collected from each well and stored at -30°C until use. Cytokine determination was performed by mouse sandwich ELISA kits following the manufacturer's instructions. The lower detection limit for both IFN-*γ* and IL-17A was 15.62 pg/mL.

### 2.7. Statistical Analysis

To perform the analysis of the normal distribution of variables, the Kolmogorov-Smirnov test was applied. The variables that presented normal distribution were shown in mean and standard deviation. The variables that did not present normal distribution were exhibited as maximum and minimum median. For immunomodulatory activity, the Wilcoxon analysis was performed using GraphPad Prism software version 6. Results were considered significant when p <0.05.

## 3. Results and Discussion

### 3.1. Characterization by TLC

Phytochemical characterization by TLC indicated the presence of coumarin, characteristic of the species, in both JPA and JPO extracts, with flavonoid compounds in JPA, due to the presence of yellow/orange bands. In JPO, in addition to the presence of flavonoids, cinnamic derivatives and steroids were observed. The presence of condensed tannins was not evidenced in any extracts (data not shown). Interestingly, other phytochemical studies of* J. pectoralis* leaves revealed the presence of compounds such as coumarin, umbelliferone, ortho-methoxylated glycosyl flavones, justicidin B, saponins, and tannins [20, 21].

### 3.2. Characterization by HPLC

**Figure 1:**
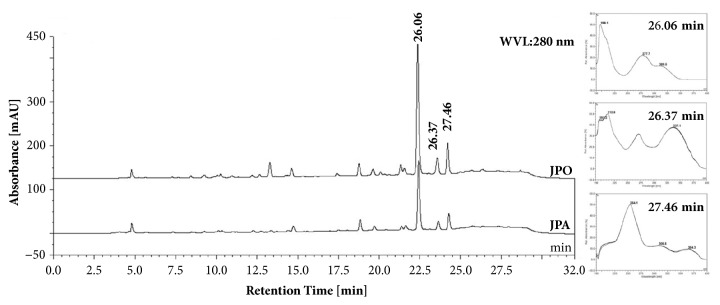
Chromatographic profile of JPA and JPO and UV-sprectra of peaks related (coumarin, apigenin, and ellagic acid). Detection at 280 nm.* Justicia pectoralis* aqueous extract (JPA);* Justicia pectoralis* organic (JPO) extracts.

Chromatograms from the HPLC analysis of the JPA and JPO can be seen in Figure 1. The same chromatographic profile was evidenced for both extracts. In both, the presence of coumarin, considered the marker of the species, with retention time (tR) equal to 26.06 min, was detected. In addition, a peak of the flavonoid apigenin was demonstrated, with tR at 26.37 min. The presence of ellagic acid was also detected, with tR = 27.46 min. The peaks were confirmed by coinjection of the standards and samples (spiked samples) and by purity analysis of peaks (> 95% and 100% at tR), verified in the chromatograms obtained with the photodiode array detector. The contents of coumarin, apigenin, and ellagic acid were calculated, based on the equation of the line obtained for the respective standards (coumarin: y = 2.0508x - 5.1038; R^2^= 0.9939; apigenin: y = 0.9054x + 3.2589; R^2^ = 0.9983; ellagic acid: y = 5.110x – 8.837; R^2^ = 0.9978). Quantities of 0.115 (0.99%) of coumarin, 0.014 (1.17%) of apigenin, and 0.015 (0.09%) of ellagic acid in JPA were measured. For JPO, the contents of this metabolites were 0.307 (0.71%), 0.030 (0.62%), and 0.016 (0.06%), respectively. The results are expressed as g%: grams per 100 g of extract and RSD: relative standard deviation.

Several studies report the coumarin quantification in aerial parts of extracts of* J. pectoralis* by HPLC [2, 22]. Coumarin (1,2-benzopyran) is one of the major secondary metabolites present in the leaves and aerial parts of* J. pectoralis* and one of the major components responsible for the biological activities attributed to this plant [23]. In the present study, the coumarin content was higher in JPA than in JPO. Compared with scientific data, variation in HPLC coumarin and other contents in leaf samples or aerial parts of* J. pectoralis* is associated with different sources of variation such as seasonality and geographic origin, in addition to conditions of plant material processing and preparation of extracts, as observed by Chanfrau and Ferrada [24].

The quantitative chromatographic analysis of ellagic acid performed for the samples used in this study revealed the same profile of coumarin contents where JPA had more contents than JPO. Other metabolites, as saponins and 3-(2-hydroxyphenyl) propionic acid, were reported presenting in* J. pectoralis* and were also quantified in extracts from leaves of this species [21, 25]. Lizcano et al. [26] also evaluated the composition of the aqueous extract of* J. pectoralis* leaves and revealed the presence of phenols and flavonoids and these compounds can contribute to the biological activities observed here.

### 3.3. Cytotoxic and Antineoplastic Profile

Regarding the cytotoxic activity, the results of the MTT and trypan blue tests showed that JPA and JPO did not exhibit cytotoxicity until 100 *μ*g/mL to PBMCs and BALB/c splenocytes (data not shown) and any minimal viability reduction was not statistically significant. Some studies include the determination of the mean lethal dose (LC_50_) of* J. pectoralis* extracts in larvae of* Artemia salina*, an alternative test for cytotoxicity determination. Parra et al. [27] showed that LC_50_ of the hydroalcoholic extract of this plant's aerial parts was 60.15 *μ*g/mL (values below 1000 *μ*g/mL are considered bioactive); that is, the extract presented bioactivity. Another study that also reports the bioactivity of* J. pectoralis's* aerial parts ethanolic extract in* Artemia salina* evaluated concentrations from 1 to 1000 *μ*g/mL. In this study, the extract did not present bioactivity (LC_50_> 1000 *μ*g/mL) against the microcrustacean [28], presumably due to the solvent used.

**Figure 2:**
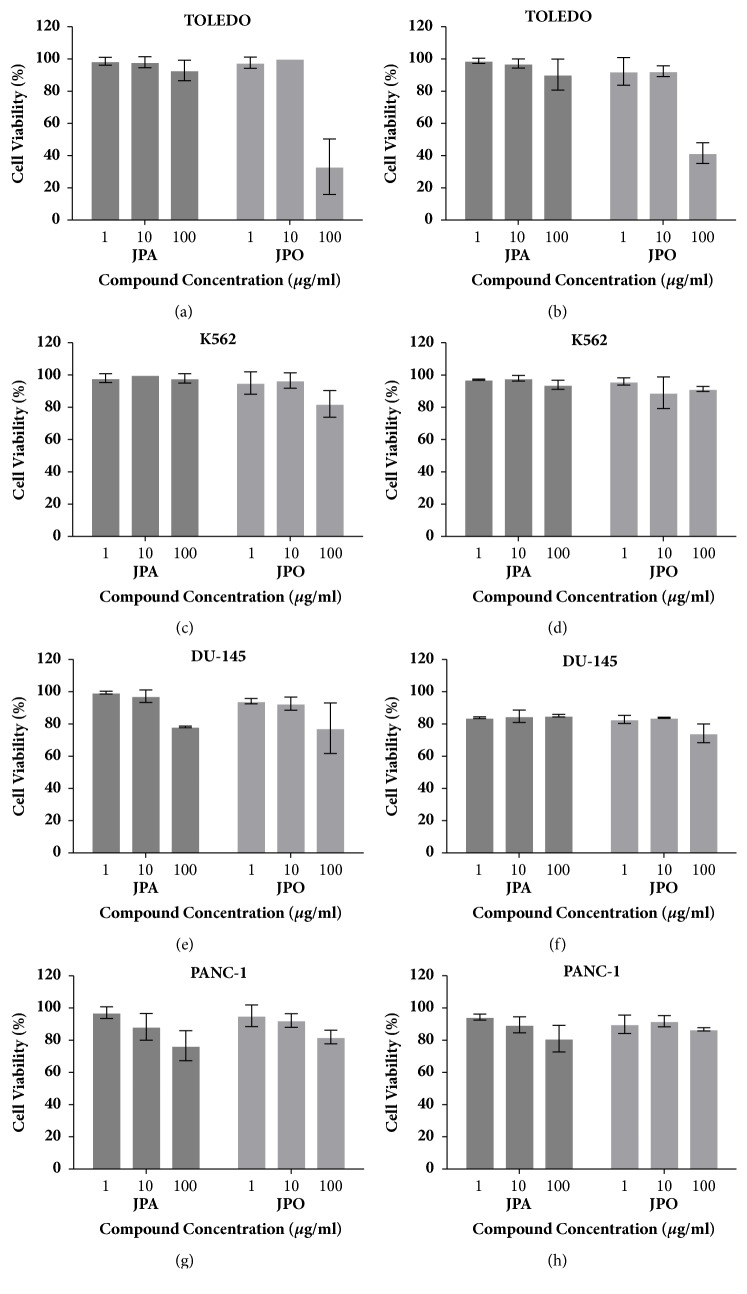
*Justicia pectoralis *cytotoxic evaluation against neoplastic cell lines TOLEDO, K562, PANC-1, and DU-145 at 1, 10, and 100 *μ*g/mL of JPA and JPO. MTT test are shown at (a), (c), (e), (g), and trypan blue are present at (b), (d), (f), and (h).

At neoplastic cell lines, MTT and trypan blue assays (Figure 2) showed that JPA exhibited cytotoxicity for PANC-1 at 100 *μ*g/mL (Figures 2(g) and 2(h)). However, at this concentration, we did not find an IC_50_ value. Despite this, JPO showed significant cytotoxicity (p<0.05) against TOLEDO at 100 *μ*g/mL (Figures 2(a) and 2(b)). The IC_50_ to TOLEDO cell line was 57.59 ± 1.03 *μ*g/mL with MTT methodology and 69.44 ± 8.08 *μ*g/mL when using the trypan blue assay. Amsacrine IC_50_ to TOLEDO and K562 were 0.5±0.6 *μ*M and 0.9±0.2 *μ*M, respectively. To DU-145, doxorubicin IC_50_ was 6.8±0.7 *μ*M and for PANC-1 Gemcitabina was used as a positive control (IC50 was 5.5±1.7 *μ*M).

There are few studies that describe anticancer properties for* J. pectoralis*. However, Joseph et al. [20] isolated a lignan, Justicidin B, from the* J. pectoralis* whole plant and found cytotoxicity against a murine leukaemia and bronchial epidermoid carcinoma cell line. In contrast, other* Justicia* species are related to anticancer potential, such as* Justicia ciliate *Jaqc. that inhibit human cervical carcinoma growth [29],* Justicia spicigera *Schltdl. that interfere in T-47D and HeLa human cell line growth [30–32], and* Justicia rhodoptera* which acts against the human ovarian cancer cell line [33].

In JPO we found the presence of coumarin and ellagic acid in its composition. Recently, because it possesses minimum side effects along with multidrug reversal activity and specially an anti-inflammatory activity, studies about coumarin derivatives as potential anticancer agents have been developed [34–36]. Additionally, ellagic acid appears to exhibit an antineoplastic action on antimetastatic breast cancer [37] and antimetastatic potential of ovarian neoplasia [38]. The ellagic acid activity against lymphoma-bearing mice was also reported [39, 40] and our results with JPO activity against TOLEDO can be associated with the presence of coumarin and ellagic acid.

### 3.4. Evaluation of Antibacterial Activity

JPO showed that MIC is equal to 500 *μ*g/mL against* Acinetobacter baumanii* and* Klebsiella pneumoniae* ESBL, indicating a bacteriostatic effect. Compared to the other bacteria isolates, the two extracts had MICs higher than 500 *μ*g/mL, indicating that they did not present a bactericidal or bacteriostatic effect. Acinetobacter is a genus of nonfermentative gram-negative bacteria that have minimal nutritional requirements and can survive in several types of aqueous environments. This group, especially the* A. baumanii* strain, is responsible for an increasing number of hospital infections, especially acquired in intensive care units (ICUs) [41]. Klebsiella pneumoniae is a Gram-negative bacillus present in the gastrointestinal tract of healthy individuals. It is also an important pathogen of nosocomial infections resistant to all cephalosporins such as ESBL, causing outbreaks in hospitalization units of critical patients [42].

Our results indicated that the aqueous extract of* J. pectoralis* did not present an inhibitory action against the eight selected bacterial strains. Martín-Viaña et al. [43] developed syrup from the dry extract of* J. pectoralis* and subjected the samples to the contamination by* Staphylococcus aureus*,* Pseudomonas aeruginosa*,* Escherichia coli*,* Candida albicans*,* Aspergillus niger*, and* Bacillus subtilis*, whose number of viable organisms was estimated in colony formation units (CFU). After 28 days, these authors detected a content of 10^2^ CFU/mL for bacteria and less than 10 CFU/mL for fungi, below the minimum limits for a sample to be considered contaminated. Furtado et al. [44] tested the aqueous extract of the leaves of* J. pectoralis* against* E. coli*,* S. aureus* and* K. pneumoniae*, Gram-positive, and Gram-negative strains by disk diffusion test. The extract showed no inhibitory action in any of the species at all concentrations (100, 50, and 25 mg/mL).

### 3.5. Modulation of IFN-*γ* and IL-17A Secretion

**Figure 3:**
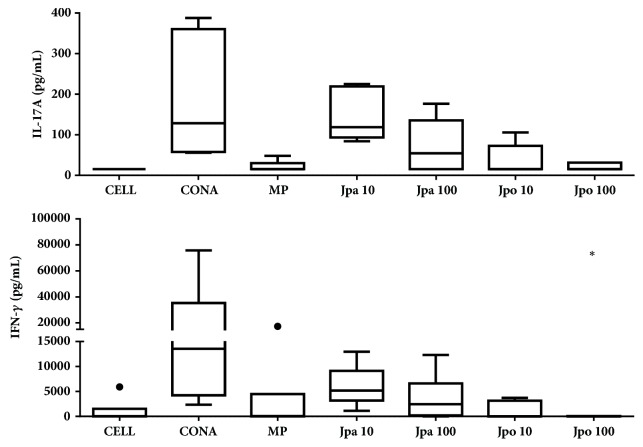
Cytokines titration in culture supernatant of Balb/c splenocytes treated with aqueous (JPA) and organic extract (JPO) of* Justicia pectoralis* at 10 and 100 *μ*g/mL. (a) IFN-*γ* levels; (b) IL-17A levels (UC = untreated control, CONA = concanavalin A (5 *μ*g/mL), and MP = methylprednisolone 100 *μ*M). *∗* p < 0.05. ● represents outliers.

Regarding the evaluation of cytokine modulation, JPO presented better results than JPA in reducing INF-*γ* and IL-17A. JPO decreased levels of IL-17A (15.62 pg/mL [79.54 pg/mL-15.62 pg/mL]) relative to the ConA-stimulated cells (128.63 pg/mL [387.76 pg/mL - 55.79 pg/mL]) (Figure 3(a)). Analysing IFN-*γ* levels, JPO decreased this cytokine more than MP (15.62 pg/mL [62.5 pg/mL-15.62 pg/mL]), 100 *μ*g/mL (p=0.03) (Figure 3(b)). The anti-inflammatory activity of this species was measured in other models, such as the one by Locklear et al. [45], who investigated the activity of methanolic extract of the* J. pectoralis* aerial parts against the symptoms of menopause and dysmenorrhea in trials of COX-2 inhibition. The extract inhibited the catalytic activity of COX-2 (IC_50 _= 4.8 *μ*g/mL).

Another study investigated the anti-inflammatory and antihistaminic activity of* J. pectoralis* leaves' aqueous extract in guinea pigs sensitized with ovalbumin (OVA). The extract blocked the effect of contraction produced by histamine in the airways [46]. The standardized extract of coumarin from the aerial parts' hydroalcoholic extract of this plant was also tested to evaluate the antiasthmatic properties in male rats previously sensitized by OVA. The hyperresponsive phenotype of tracheal tissues caused by OVA decreased with the administration of the standardized extract. Furthermore, the extract abolished the OVA-induced increase in the IL-1*β* and TNF-*α* level in the bronchoalveolar fluid and weakened the changes in the expression of canonical transient receptor protein genes. These findings evidenced that* J. pectoralis* presents antiasthmatic properties in the experimental model of reproductive hyperresponsiveness [2]. Our work corroborates with the research of these authors regarding the anti-inflammatory activity of this plant, despite using another type of organic extract to the tested cytokines.

In other* Justicia* species, it is possible to identify immunomodulatory characteristics, as described by Kumar et al. [47] when reporting the inhibition of paw edema after treatment with fractions from ethyl-acetate fraction of* Justicia gendarussa*. In another study using the carrageenan and formalin-induced paw edema models, it was found a dose-dependent response after treatment with methanolic extract of* Justicia secunda* leaf at carrageenan paw edema models, indicating the significant inhibition after 2 h, 3 h, and 24 h treatment at 0.1, 0.2, and 0.4 g/kg [48]. The anti-inflammatory action of* Justicia acuminatissima* leaves in animal models was also reported and showed that the aqueous extract caused inhibition of inflammatory pain in a formalin-induced paw licking test at 30, 100, and 300 mg/kg. In addition, the aqueous extract presented statistically significant inhibition of NO_2_^−^ formation, an oxidized form of NO released by LPS-activated macrophages, where this event is an important molecular mechanism implied in the inflammatory response [49].

Aqueous and organic extracts showed no significant toxicity in PBMC. JPO, in turn, further modulated the cytokines and proved to be more bacteriostatic.* J. pectoralis* therefore appears to have a strong anti-inflammatory action, and this may be associated with the various applications of this plant in folk medicine given the diseases for which the species is indicated to be of inflammatory basis.

## 4. Conclusions

Coumarin and ellagic acid were the major components of JPA and JPO and probably contributed to the anti-inflammatory activity evidenced in this work. Lower toxicity to nontransformed cells and cytotoxicity to neoplastic cells were observed. The organic extract showed bacteriostatic activity against* Acinetobacter baumannii* and* Klebsiella pneumoniae*. Therefore,* Justicia pectoralis* is a potential candidate for novel in vivo studies to evaluate its anti-inflammatory properties.

## Data Availability

The data used to support the findings of this study are included within the article.

## Supplementary Materials

Supplementary Materials Mobile phase, reagents, and standards used to phytochemical characterization of Justicia pectoralis crude extracts.
