# Supplementary material for: Organic Extract of Justicia pectoralis Jacq. Leaf Inhibits Interferon-γ Secretion and Has Bacteriostatic Activity against Acinetobacter baumannii and Klebsiella pneumoniae
Source: Evid Based Complement Alternat Med. 2018 Aug 23;2018:5762368. doi: 10.1155/2018/5762368 (PMC6126107; doi:10.1155/2018/5762368)
Supplement: Supplementary Materials — Mobile phase, reagents, and standards used to phytochemical characterization of Justicia pectoralis crude extracts. [file 5762368.f1.pdf]

Supplementary Material

Table S1. Mobile phase, reagents and standards used to phytochemical characterization of *Justicia pectoralis* crude extracts.

**Metabolites Class**	**Mobile Phase**	**Reagents**	**Standards**
Hydrolysable tannins	90:5:5	NEU + PEG	Gallic Acid and Ellagic Acid
Flavonoids	90:5:5	NEU + PEG	Quercetin and Rutin
Cinnamic derivatives	90:5:5	NEU + PEG	Caffeic Acid and Chlorogenic Acid
Condensed tannins	90:5:5	Vanilin chloride	Catechin
Coumarin	50:50:50	KOH + Δ	Coumarin
Terpenes and Steroids	70:30	Lieberman-Burchard + Δ	β-Sitosterol

Systems: 90:5:5 – ethyl acetate : formic acid : water; 50:50:50 - ethyl ether: ethyl acetate: acetic acid 10% (saturation); 70:30 – Toluene: ethyl acetate; KOH: Potassium hydroxide; Δ – heating. NEU: 2-Aminoethyl diphenyl borate; PEG: polyethylenoglicol.
